# Upregulation of long intergenic noncoding RNA 00673 promotes tumor proliferation via LSD1 interaction and repression of NCALD in non-small-cell lung cancer

**DOI:** 10.18632/oncotarget.8338

**Published:** 2016-03-24

**Authors:** Xuefei Shi, Chenhui Ma, Qingqing Zhu, Dongmei Yuan, Ming Sun, Xiaoling Gu, Guannan Wu, Tangfeng Lv, Yong Song

**Affiliations:** ^1^ Department of Respiratory Medicine, Jinling Hospital, Nanjing University School of Medicine, Nanjing, China; ^2^ Department of Biochemistry and Molecular Biology, Nanjing Medical University, Nanjing, China; ^3^ Department of Respiratory Medicine, Huzhou Central Hospital, Huzhou, China

**Keywords:** linc00673, proliferation, LSD1, NCALD, NSCLC

## Abstract

Despite improvements in diagnostics and treatment of non-small cell lung cancer (NSCLC), it remains the leading causes of cancer-related mortality worldwide. In more recent years, mutiple lines of evidence have highlighted long noncoding RNAs (lncRNAs) serve as novel class of regulators of cancer biological processes, including proliferation, apoptosis and metastasis. LncRNAs serve as a novel class of regulators of cancer biological processes in cancer, but little is known of their expression and potential functions in NSCLC. We identified an oncogene, linc00673, whose expression level was upregulated by bioinformatics analyses and qRT-PCR analyses in NSCLC. The effects of linc00673 on tumor progression were investigated *in vitro* and *in vivo*. Linc00673 knockdown significantly inhibited cell proliferation and colony-forming ability, and suppressed S-phase entry *in vitro* and shRNA linc00673 mediated knockdown significantly inhibit tumor growth *in vivo*, meanwhile, linc00673 overexpression increased tumor cell growth. Analysis of RNAseq data revealed linc00673 could modulate the transcription of a large amount of genes including oncogene and tumor suppressor gene, so we investigated the role and regulatory mechanism of linc00673 in NSCLC proliferation. Further mechanistic analyses indicated that the oncogenic activity of linc00673 is partially attributable to its repression of NCALD through association with the epigenetic repressor LSD1. Taken together, these findings suggested that linc00673 could play crucial role in NSCLC progression and might be a potential therapeutic target for patients with NSCLC.

## INTRODUCTION

In spite of improvements in cancer surgeries, radiotherapy, and anti-cancer drugs in lung cancer, which remains the leading cause of cancer-related death worldwide. According to the American Cancer Society (ACS), it is expected to account for 28% of all male cancer deaths and 26% of all female cancer deaths in 2015 [1]. Non-small-cell lung cancer (NSCLC) is the most prevalent histological type of lung cancer and can be categorized into two common subtypes, adenocarcinoma and squamous cell carcinoma [2–3]. The primary reason for such a high mortality rate is its cell-sustained proliferation and metastatic potential [4]. Undoubtedly, understanding the molecular mechanisms associated with malignant proliferation is crucial for establishing more effective approaches for NSCLC treatment that can improve patient outcomes.

Although research has focused on the role of protein-coding genes in the pathogenesis of human tumors in the past few decades, non-coding RNAs (ncRNAs), including microRNAs and long non-coding RNAs (lncRNAs) have received increased attention with development of whole genome and transcriptome sequencing technologies [5]. LncRNAs are a class of non-protein-coding RNA molecules which are longer than 200 nucleotides [6]. The ENCODE Consortium has elucidated that human transcription generates thousands of lncRNAs and they can be further categorized as genic (exonic, intronic, overlapping) or intergenic lncRNAs according to their location with respect to the nearest protein-coding transcripts [7]. The idea that lncRNAs are not just cloning artifacts or transcriptional noise but rather important supplements to proteins or crucial regulators in complex networkscontinues to advance in the scientific community [8–9]. In fact, many lncRNAs have been shown to express in various developmental stages-, tissues-, and organ-specific patterns, as well as participate in a wide spectrum of biological processes, such as cell differentiation, embryonic development and carcinogenesis [10–12]. More importantly, there is evidence linking the dysregulation of lncRNAs with various cancer types which implies that lncRNAs might function as potential onco- or tumor-suppressor RNAs [13–14]. In our previous study, we identified two lncRNAs involved in NSCLC tumorigenesis. We determined that GAS5 (growth arrest-specific transcript 5) was not only dysregulated in numerous cancers [15–16] but specifically down-regulated in NSCLC. GAS5 increased tumor cell growth arrest and induced apoptosis through P53-dependent and P53-independent pathways [17]. In addition to GAS5, HNF1A-AS1 was up-regulated in esophageal adenocarcinoma and that it could induce the expression level of lncRNA H19 and lead to an increase in cell proliferation, migration and invasion, Nevertheless, we discovered another underlying molecular mechanism of HNF1A-AS1 which plays a key role in lung adenocarcinoma tumorigenesis. HNF1A-AS1 might bind to DNMT1 resulting in reducing the expression level of E-cadherin [18–19]. In this regard, identifying cancer-associated lncRNAs the associated molecular mechanisms are necessary for understanding progression and establishing better treatment of NSCLC.

Neurocalcin delta (NCALD) is a member of the visinin-like subfamily of EF hand calcium-binding proteins [20]. Subsequent studies show that NCALD might be involved in the pathogenesis of human cancer. A study determined varying gene expression profiles in ovarian cancer patients' whole blood cell mRNA [21]. It was determined that NCALD mRNA was down-regulated in the group with poor prognosis, having advanced stage and poorly differentiated tumors which implied NCALD a prognostic biomarker specific to these ovarian cancer patients [22]. Other studies have documented down-regulation of NCALD in asbestos-related lung cancers [22], however, so far very little is known about the specific function role of this gene in NSCLC.

In the current study, we screened for the novel candidate lncRNAs responsible for the progression of NSCLC. We identified long intergenic non-coding RNA 00673 (linc00673) was the most remarkably up-regulated lncRNA in NSCLC tissues, compared with adjacent normal lung tissue. We then focus on the functional roles and further molecular mechanism of linc00673 in NSCLC cell lines. The results showed that linc00673 could promote NSCLC cell proliferation by epigenetically inhibiting NCALD expression by binding to LSD1.

## RESULTS

### The expression profile of lncRNA in NSCLC

**Figure 1:**
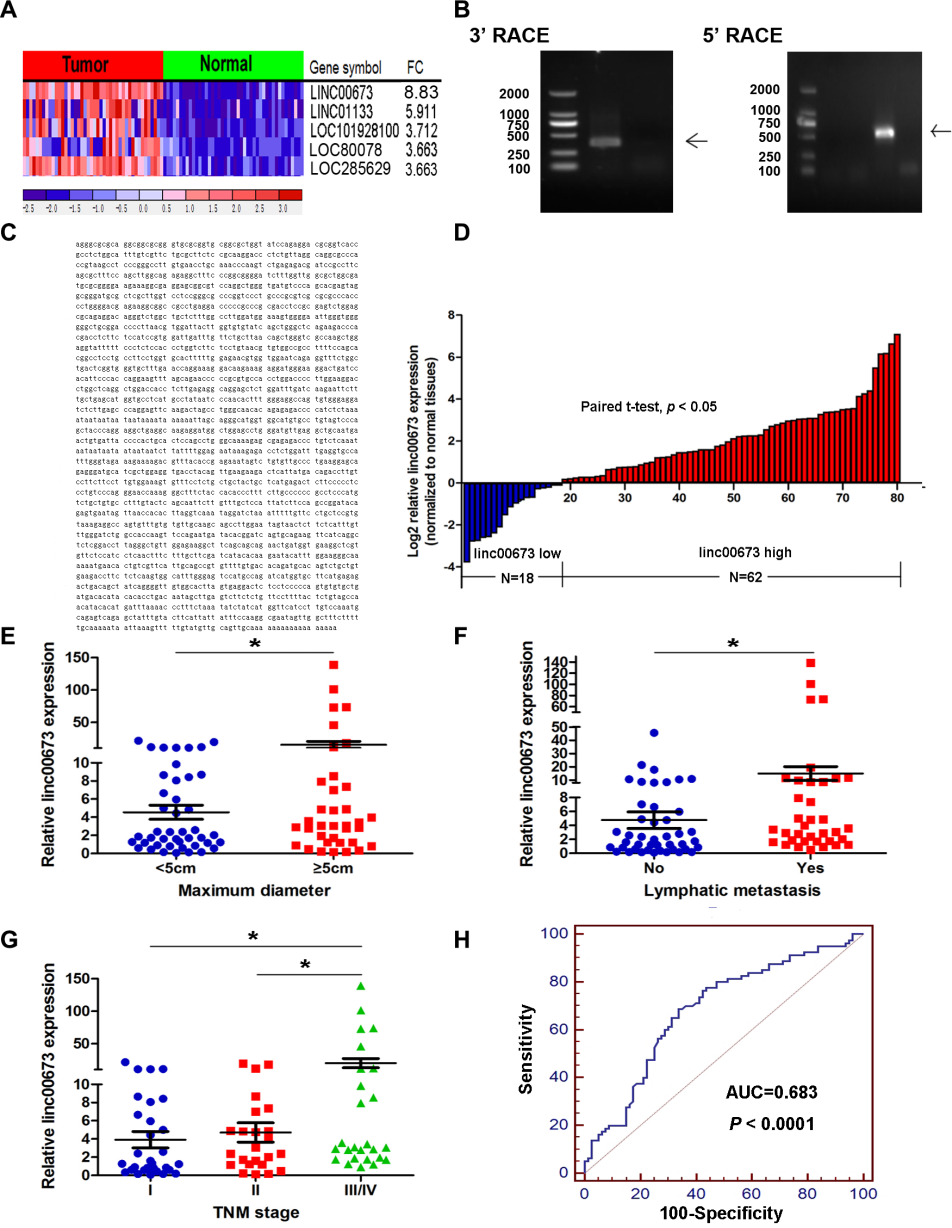
Higher linc00673 expression levels in NSCLC (**A**) A heatmap representation of the five lncRNAs with the most upregulation across NSCLC and normal lung tissues in GSE18842. (**B**) Agarose gel electrophoresis analysis of the PCR products (indicated by black arrows) that were generated in 3′ (left) and 5′ (right) RACE which covered the 3′ and 5′ ends of the linc00673 in A549 cells. (**C**) The full sequence of linc00673 was confirmed by RACEs and the result of RACEs was consistent with the sequences published in NCBI database (NR_036488.1). (**D**) Quantitative real-time PCR analysis of linc00673 expression level in 80 NSCLC tissues and corresponding adjacent non-tumor tissues (two-tailed student's *t* test, *P* < 0.05). Expression levels were shown as log2-fold change to match non-tumor tissues. Red column represented overexpression and blue column represented down-regulation. (**E**, **F** and **G**) The relationship between linc00673 expression and clinicopathological parameters (such as maximum diameter, lymphatic metastasis and TNM stage) was shown. Data are shown as the mean ± s.d. Based on at least three independent experiments. *P < 0.05, **P < 0.01. (**H**) The ROC curve for prediction of NSCLC based on the expression level of linc00673, using paired adjacent non-tumorous tissues as a control.

In an attempt to identify novel oncogenic lncRNAs in NSCLC progression, a microarray dataset (GSE18842) was used to analyze differentially expressed lncRNAs between lung tumors samples and corresponding nontumor samples. The microarray analysis revealed 27 upregulation lncRNAs and 72 downregulation lncRNAs (Supplementary Figure S1A and Supplementary Table S1 Sheet 3). The most upregulation lncRNAs were linc00673, linc01133, Loc101928100, Loc80078 and Loc285629 in NSCLC (Figure 1A). Notably, recently studies have shown linc01133 to be upregulated in lung squamous cell cancer, and also that the expression level of lin01133 is indicative of patient survival [23]. To investigate the role of lncRNAs in NSCLC tumorigenesis, we selected linc00673, to which the expression was most significantly different, for further investigation.

We first identified the full poly (A)-positive sequence of linc00673 through rapid amplification of cDNA ends (RACE) (Figure 1B) and performed PCR analysis to confirm the gene size of linc00673 (2275 bp, Figure 1C). In addition, txCdsPredict, created by UCSC, was used to calculate its protein coding potential, and a score was assigned based on its coding potential, we considered the transcript as noncoding RNA when the score was less than 800 [24]. The txCdsPredict score for linc00673 was 454, indicating that linc00673 has no protein-coding potential.

### Upregulation of linc00673 expression in NSCLC tissues

In order to ascertain whether linc00673 was differentially expressed in the NSCLC tissues, the expression level of linc00673 was quantified by qRT-PCR in 80 paired clinical NSCLC tissues and correspondingnormal tissues. Results determined that betweentissue samples, linc00673 was up-regulated (9.37-fold) in clinical NSCLC tissues (*P* < 0.05, Figure 1D). We also evaluated the correlation of linc00673 expression with patients' clinicopathological parameters (i.e., maximum diameter, lymphatic metastasis or TNM stage) to assess its clinical significance. Our results showed that larger tumors, with lymph node metastasis, or more advanced tumors, had higher linc00673 expression levels (Figure 1E, 1F and 1G). Nevertheless, there was no significant relationship between linc00673 expression and other clinical characteristics, such as age, gender, differentiation and smoking history (*P* > 0.05, Table 1).

**Table 1:** Correlation between linc00673 expression and clinicopathological parameters of NSCLC

Clinicopathological parameters	N of cases	Log2 relative linc00673 expression		
		Low	High	*P*-value^a^
Age (years)				0.382
< 65	39	17	22	
≥ 65	41	22	19	
Gender				0.214
male	58	31	27	
female	22	8	14	
Differentiation				0.625
well, moderate	57	29	28	
poor	23	10	13	
Tumor size (maximum diametercm)			0.015^b^	
< 5 cm	44	27	17	
≥ 5 cm	36	12	24	
Primary location				0.111
left lung	33	20	13	
right lung	47	19	28	
Histology type				0.256
adenocarcinoma	47	20	27	
squamous carcinoma	33	19	14	
Smoking history				0.187
smokers	43	24	19	
never smokers	37	15	22	
Lymph node metastasis				0.047^b^
positive	36	13	23	
negative	44	26	18	
TMN stage				0.033^b^
I/II	54	31	23	
III/IV	26	8	18	

a Chi-square test.

* *P* < 0.05

Additionally, we produced a receiver operating characteristic curve (ROC curve), using corresponding normal tissues as control. The cutoff value for distinguishing NSCLC tissues from normal tissues was 7.75 (ΔCt). The sensitivity and specificity were 68.75 and 66.25, respectively, and the area under the ROC curve was 0.683 (95% confidence interval: 0.605 to 0.755, *P* < 0.0001, Figure 1H). These data demonstrate that linc00673 might be an oncogene in the context of NSCLC progression and may potentiallyserve as diagnostic biomarker for NSCLC.

### Modulation of linc00673 expression in NSCLC cells

**Figure 2:**
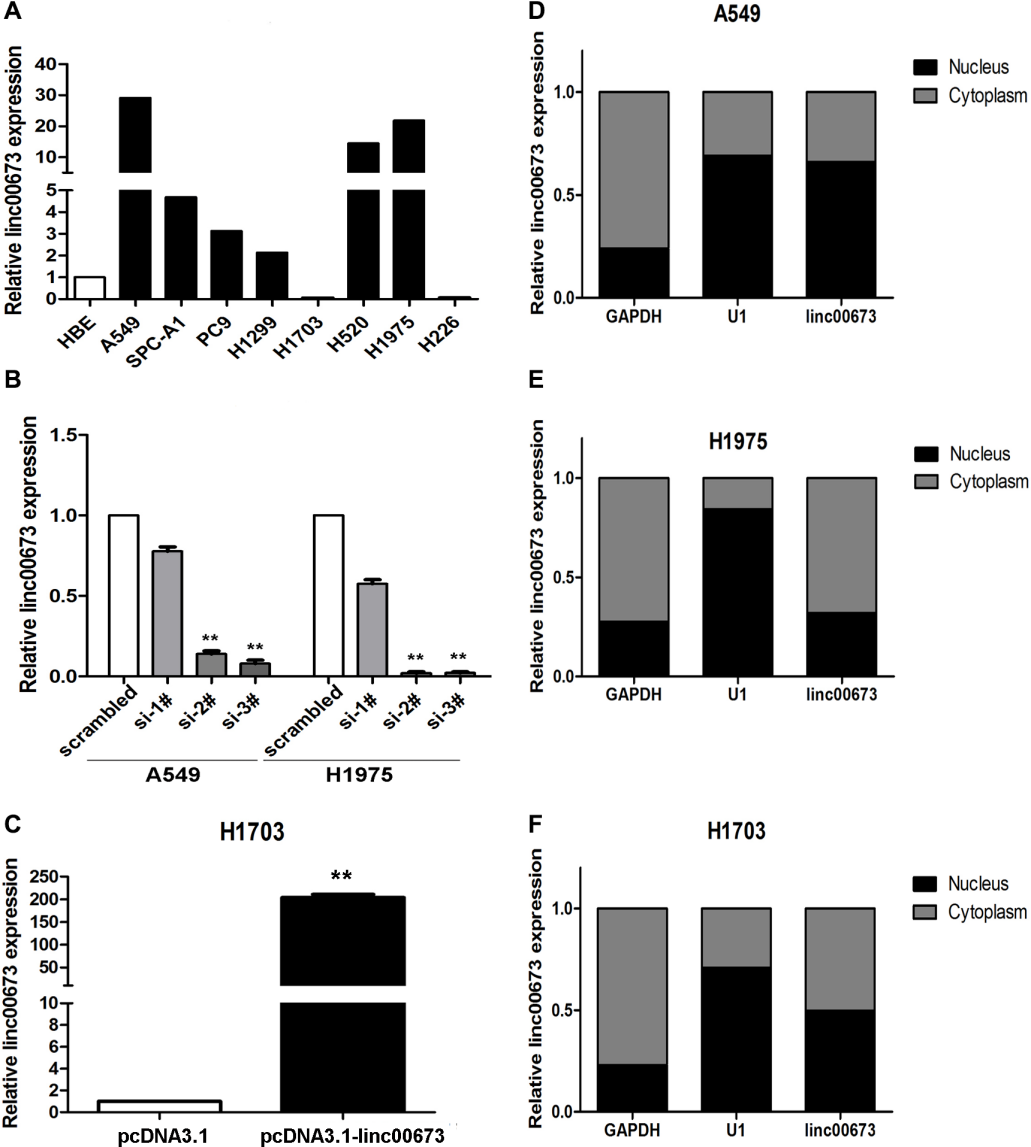
Linc00673 expression increased in NSCLC cells (**A**) qRT-PCR analysis of linc00673 expression level in 8 NSCLC cell lines (3 squamous carcinoma cell lines and 5 adenocarcinoma cell lines) and 1 human bronchial epithelial cell (HBE). The expression levels are normalised to HBEs. (**B**) qRT-PCR analysis of linc00673 expression level in A549 and H1975 transfected with three discrete chemically synthesized siRNAs. (**c**) qRT-PCR analysis of linc00673 expression level in A549 and H1975 transfected with pcDNA3.1-linc00673 vector. (**D**, **E** and **F**) Linc00673 expression levels in different subcellular fractions in A549 (D), H1975 (E) and H1703 (F) cells were detected by qRT-PCR. Black range indicates nuclear fraction, Gray indicates cytoplasmic fraction. Data are shown as the mean ± s.d. Based on at least three independent experiments. *P < 0.05, **P < 0.01.

We performed qRT-PCR analysis to determine the expression level of linc00673 in 8 human NSCLC cell lines which include both squamous carcinoma and adenocarcinoma. It was determined that linc00673 expression was elevated to in 6 lung cancer cell lines, whereas linc00673 expression was lower in H1703 and H226 than that in human bronchial epithelial cells (HBEs) (Figure 2A). We used three chemically synthesized siRNAs to knock down endogenous linc00673 in A549, H1975 and SPC-A1, and developed a linc00673 overexpression model using transfection of pcDNA3.1-linc00673 in H1703. At 48 h post-transfection, linc00673 expression levels were knocked down more than 80% in A549, H1975 and SPC-A1 relative to negative control transfected cells (Figure 2B and Supplementary Figure S1B). Linc00673 expression level was substantially up-regulated via pcDNA3.1-linc00673 transfection, and the efficiency of transfection for H1703 was 210-fold (Figure 2C). Furthermore, we measured linc00673 expression in nuclear and cytosolic fractions from 4 NSCLC cell lines (including A549, H1975, SPC-A1 and H1703). As shown in Figure 2B, 2E, 2F and Figure S1C, linc00673 was localized in the nucleus and cytosol, which implies that linc00673 may exert both transcription and post-transcriptional level regulatory functions in NSCLC cell lines.

### The effect of linc00673 on NSCLC cell proliferation *in vitro*

**Figure 3:**
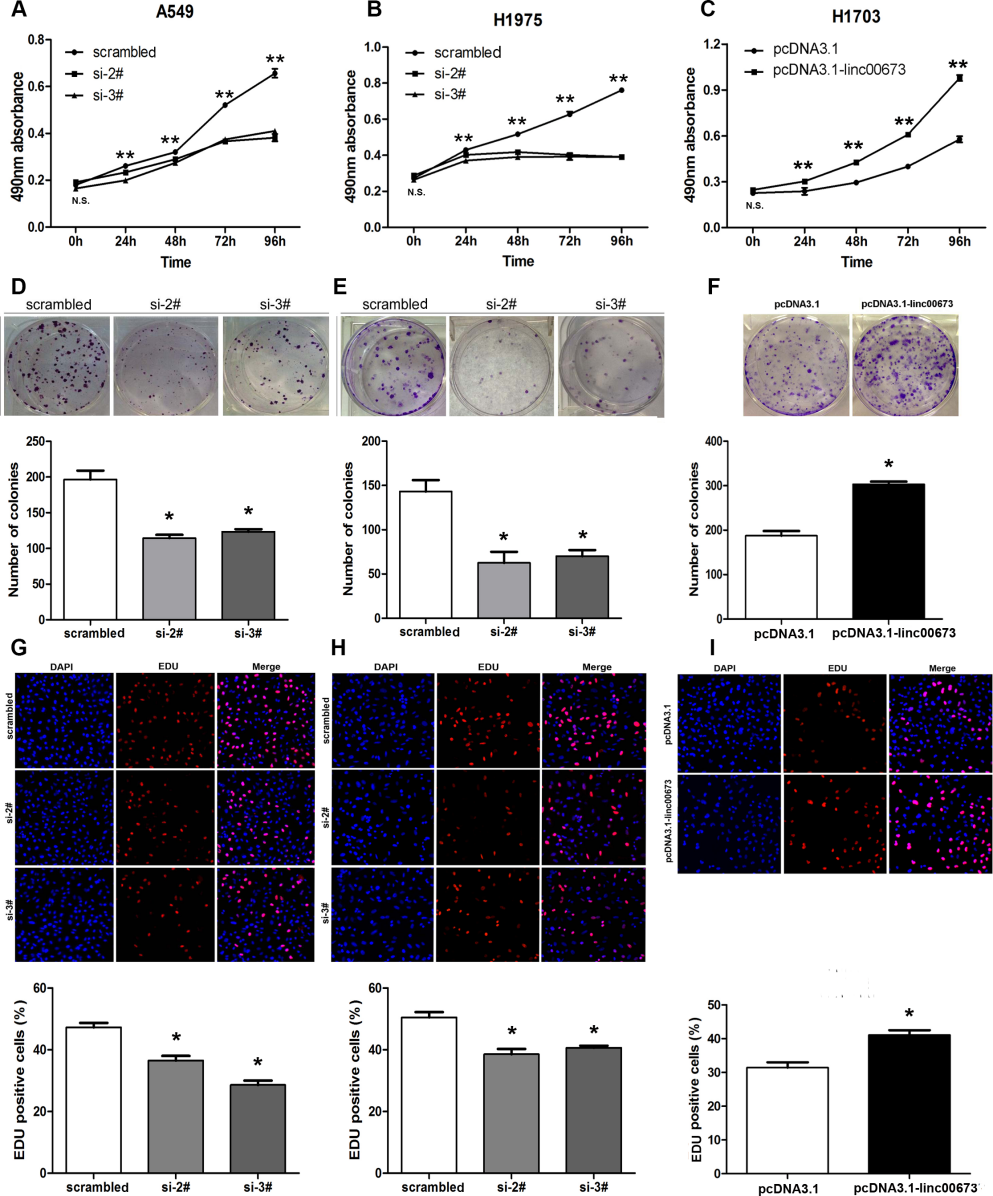
The effects of linc00673 on NSCLC cell viability *in vitro* A549 and H1975 cells were transfected with scramble or siRNA linc00673 (si-2# or si-3#), while, H1703 cells were transfected with pcDNA3.1 or pcDNA3.1-linc00673. (**A**) MTT assays were performed to determine the cell viability of transfected NSCLC cells. (**B**) A colony-forming assay was conducted to determine the proliferation of transfected NSCLC cells. The colonies were counted and captured. (**E**) EDU (red)/DAPI (blue) immunostaining assay was used to confirme the results of MTT assay and colony-forming assay. The data represent the mean ± SD from three independent experiments. *P < 0.05, **P < 0.01.

Since human lncRNAs play an important role in the spectrum of biological processes, we examined whether linc00673 was functionally involved in NSCLC tumorigenesis. MTT assays were used to determine cell viability in the NSCLC cell lines. siRNA transfection-mediated linc00673 knockdown resulted in a significant decrease in cell viability rate inA549, SPC-A1 and H1975, which tend to exhibit naturally high linc00673 expression levels (Figure 3A, 3B and Supplementary Figure S2A). Consistently, H1703 cells, which exhibit naturally low linc00673 expression, displayed a higher cell viability rate after linc00673 overexpression relative to negative control (Figure 3C). In addition, the colony-formation assay showed that a decrease in linc00673 expression also greatly attenuated the colony-forming ability of A549, H1975 and SPC-A1 (Figure 3D, 3E and Supplementary Figure S2B), while an increase in linc00673 expression enhanced colony-formation ability of H1703 (Figure 3F). These observations were further confirmed by EDU (red)/DAPI (blue) immunostaining assay (Figure 3G, 3H, 3I and Supplementary Figure S2C). Collectively, these results validated a positive role of linc00673 in promoting NSCLC cell proliferation, a crucial process in cancer progression.

### siRNA-mediated knockdown of linc00673 promotes G1 arrest through downregulation CDK6 expression

**Figure 4:**
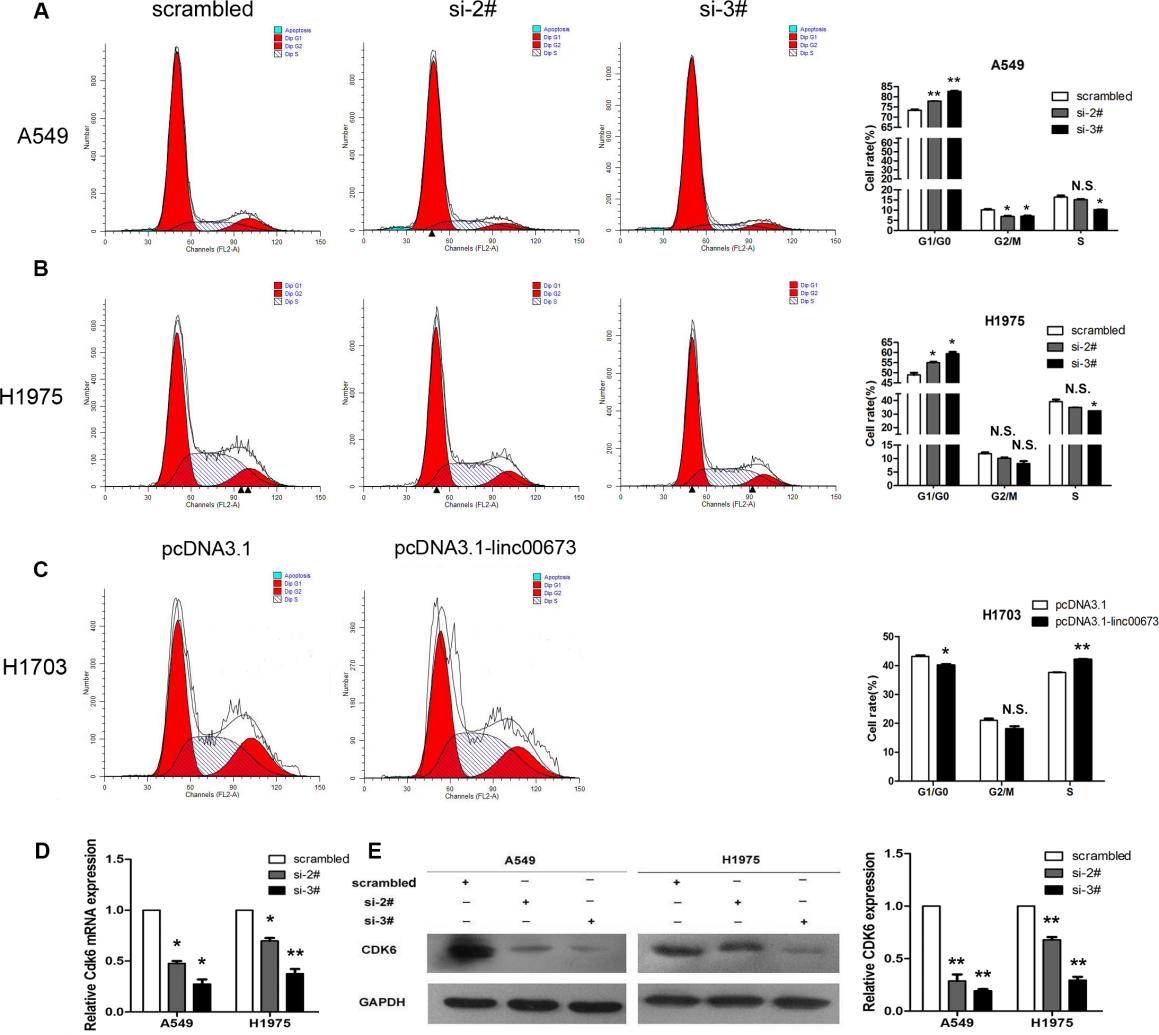
The effects of linc00673 on NSCLC cell cycle *in vitro* A549 and H1975 cells were transfected with scramble or siRNA linc00673 (si-2# or si-3#), while, H1703 cells were transfected with pcDNA3.1 or pcDNA3.1-linc00673. (**A**, **B** and **C**) Cell cycle analyses in the A549, H1975 and H1703 cell lines. Representative fluorescence activated cell sorting images and statistics based were presented. (**D**) QRT-PCR analysis of CDK6 mRNA expression level in A549 and H1975 cells. (**E** and **F**) Analysis of CDK6 protein level in A549 and H1975 cells by Western blot. GAPDH protein expression was used as an internal control. The data represent the mean ± SD from three independent experiments. *P < 0.05, **P < 0.01.

To further determine whether the impact of linc00673 on NSCLC cell growth induced cell cycle arrest, FACS technology was applied. Results revealed NSCLC cells treated with si-2# or si-3# had increased cell cycle arrest in the G0/G1 phase (Figure 4A, 4B and Supplementary Figure S2D). On the contrary, overexpression linc00673 led to a decrease in cells arrested in G0/G1 and an accumulation of cells at S phase (Figure 4C). Furthermore, we detected the expression levels of CDKs (cyclin-dependent kinases) after transfection. Compared to control cells, linc00673 downregulation significantly reduced CDK6 (cyclin-dependent kinase 6) transcript levelsand protein (Figure 4D, 4E and Supplementary Figure S2F, S2H), and did not reduced CDK2 and CDK4 protein levels (Supplementary Figure S2G). Previous studies have indicated that CDK6 is a target of TDP-43, and CDK6 expression is mediated by TDP-43 recruitment to GU-rich transcript [25]. Here, we found that linc00673 can directly bind with TDP-43 in A549 and H1975 cells by RIP analysis (Supplementary Figure S2J). Accordingly, we proposed that linc00673 might upregulate CDK6 expression by competing withTDP-43 binding sites, resulting in NSCLC cell cycle progression.

The effect of linc00673 on apoptosis was also determined by FACS technology. Results indicated that the proportion of apoptotic H1975 cells following linc00673 knockdown, versus scrambled siRNAs, remained similar (Supplementary Figure S2E). Thus, linc00673-mediated acceleration of NSCLC cell proliferation appears to be mediated by affecting the G1-S checkpoint, rather than by apoptotic pathways.

### Downregulation of linc00673 inhibits NSCLC cell tumorigenesis *in vivo*

**Figure 5:**
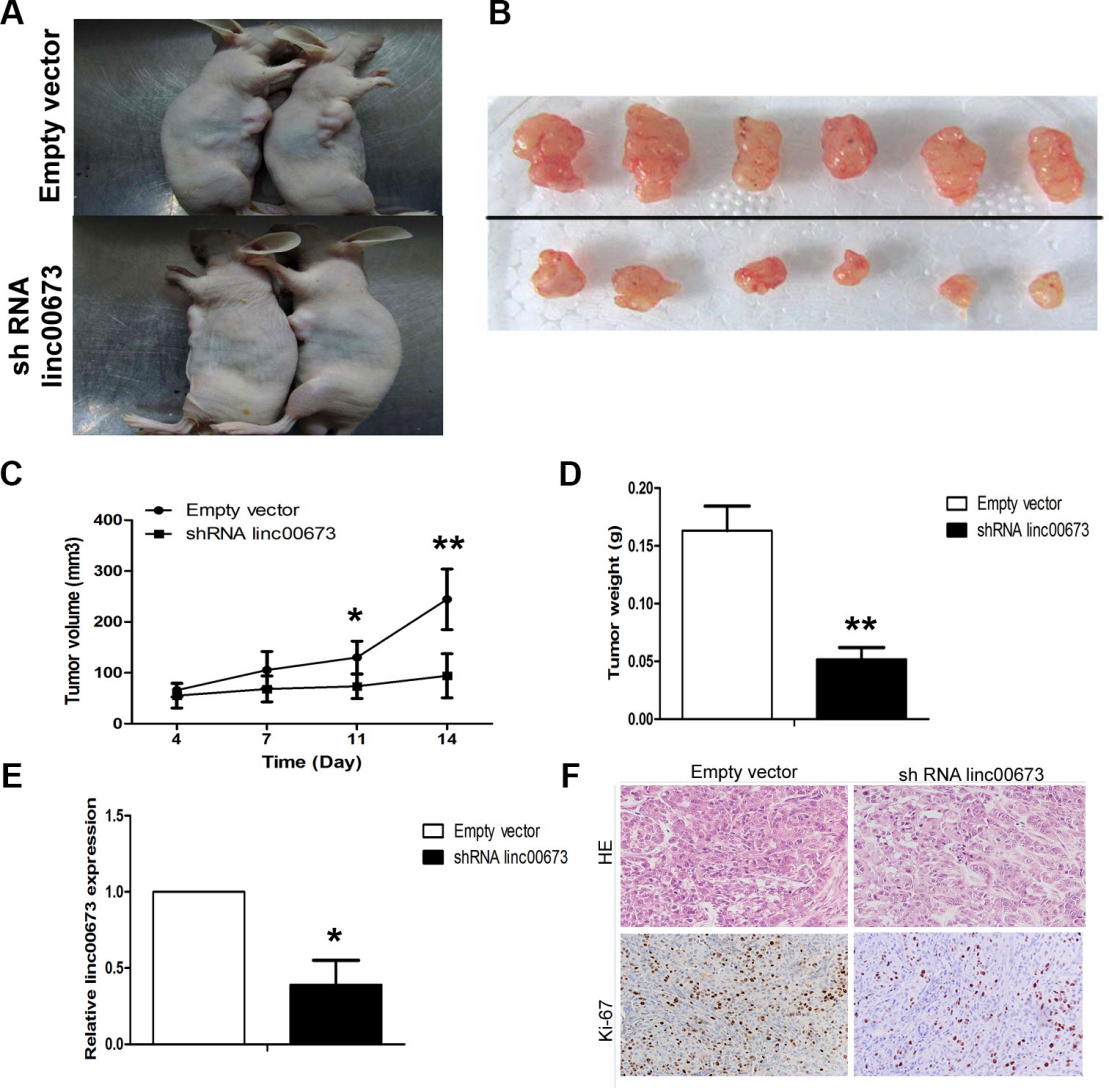
The effects on tumor growth after linc00673 downregulation *in vivo* (**A, B**) A549 cells stably transfected with shRNA lin00673 or empty vector were injected into nude mice as described in the Materials and Methods Section. Tumors before and after removal from the mice. (**C**) The tumor volume was calculated once every 3 or 4 days after injection of A549 cells stably transfected with shRNA lin00673 or empty vector. (**D**) Tumor weight when the tumors were harvested. (**E**) qRT-PCR analysis of linc00673 expression level in tumor tissues formed from shRNA linc00673 or empty vector transfected A549 cells. (**F**) Representative images (×200) of HE staining and Ki-67 immunohistochemistry of the tumor. Top, H & E staining; bottom, immunostaining. The data represent the mean ± SD from three independent experiments. *P < 0.05, **P < 0.01.

To further provide *in vivo* evidence for the oncogenic role of linc00673 in NSCLC, we used a xenograft mouse model. A549 cells stably transfected with sh linc00673 or an empty vector were subcutaneously injected into male nude mice. Four days post-injection, all mice developed detectable tumors. Linc00673 knockdown treatment dramatically decreased tumor growth, which was determined by significantly reduced tumor size and weight, relative to the control (Figure 5A–5D). Quantitative RT-PCR analysis found that the expression levels of linc00673 in tumors after shRNA linc00673 transfection were lower than those in tumors after empty vector transfection (Figure 5E). Thus, decreased linc00673 transcripts reduce the growth of established NSCLC xenografts. The HE staining showed typical characteristics of tumor cells, and the proliferation index Ki67 determined by immunohistochemical staining, was significantly decreased in shRNA linc00673-transfected tumors (Figure 5F). Based on our preliminary evidence, we highlighted an important role of linc00673 in human NSCLC, however, the mechanism(s) governing the oncogenic role of linc00673 in such thisdisease have yet to be elucidated.

### Gene expression profiling

**Figure 6:**
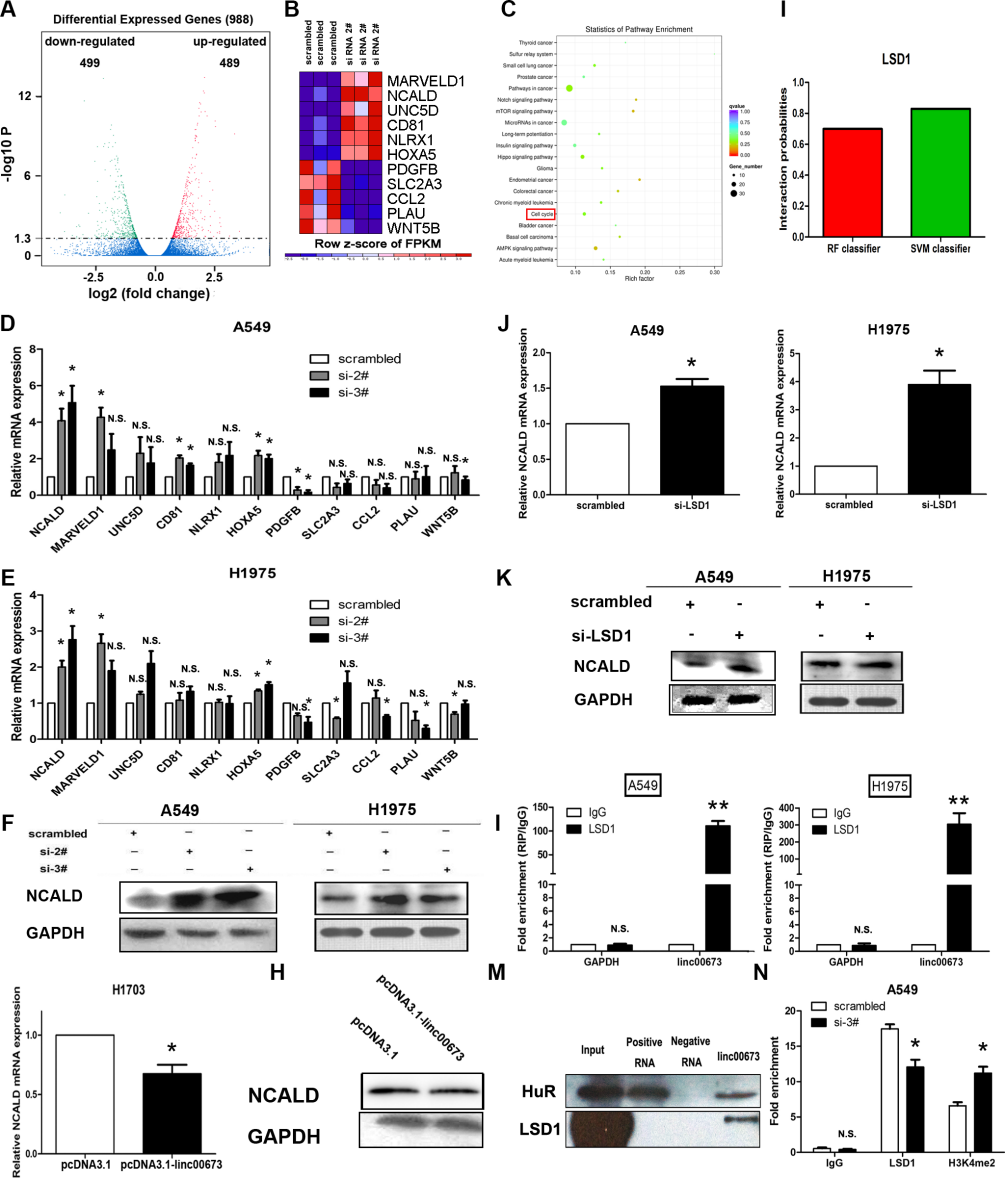
Linc00673 could directly bind LSD1 and silence NCLAD transcription (**A**) RNA transcriptome sequencing analysis was performed to analyze gene expression profiling in A549 cells following linc00673 knockdown. Volcano plot showed the all of different expressed gene. (**A**) Expression heatmap of 11 significant different expressed transcripts after the transduction of siRNA linc00673 2#. Expression profiles of the 11 probes in cells with scramble siRNA (left) and with linc00673 knockdown (right) are shown. Red and blue indicate up- and down-regulation, respectively. (**C**) KEGG pathway analysis for all genes with altered expressions between the negative control group and linc00673 knockdown group cells *in vitro*. (**D** and **E**) QRT-PCR analysis of the mRNA expression levels of tumor suppressor genes and oncogenes in control (scrambled) vs si linc00673 (si-2# and si-3#)-treated NSCLC cells. (**F**) Western blot analysis of NCALD protein level in A549 and H1975 cells. GAPDH protein expression was used as an internal control. (**G** and **H**) QRT-PCR analysis and western blot analysis of NCALD mRNA and protein level in control (pcDNA3.1) vs linc00673 overexpression. (**I**) The prediction of the interaction probabilities of linc00673 with RNA binding protein (LSD1) via RNA-Protein interaction prediction (http://pridb.gdcb.iastate.edu/RPISeq/). (**J** and **K**) QRT-PCR analysis and western blot analysis of NCALD mRNA and protein level in A549 and H1975 after LSD1 knockdown. (**L**) RIP with rabbit monoclonal anti-LSD1 and preimmune IgG from A549 (left) and H1975 (right) cell extracts. RNA levels in immunoprecipitates were determined by qPCR. Expression levels of linc00673 RNA were presented as fold enrichment in LSD1 relative to IgG immunoprecipitates. (**M**) Biotinylated linc00673 RNA pulls down the LSD1 protein detected by western blot analysis. HuR as a positive control. (**N**) ChIP–qRT-PCR of LSD1 occupancy and H3K4-me2 binding in the NCALD promoter in A549 cells treated with linc00673 siRNA (48 hours) or scrambled siRNA. The data represent the mean ± SD from three independent experiments. *P < 0.05, **P < 0.01.

In order to analyze the linc00673-associated gene transcriptional changes, we applied RNA transcriptome sequencing to assess the gene expression profiles of linc00673-depleted A549 cells and control cells. This unbiased genome-scale analysis identified 988 differentially expressed transcripts (|log2(FoldChange)| > 1 and *P* < 0.05) in NSCLC cells after linc00673 knockdown comparedto controls, including 499 downregulation transcripts and 489 upregulation transcripts (Figure 6A and Supplementary Table S1 Sheet4). Furthermore, to investigatethe functional processes that were affected by linc00673-mediated transcriptional regulation, KEGG (Kyoto Encyclopedia of Genes and Genomes) analysis was performed. We determined that cell cycle progression was involved in the affected functional processes in linc00673-depeleted cells (Figure 6C), which was consistent with our data. Using qRT-PCR, we confirmed representative genes (Figure 6B) which were identified as oncogenes or tumor suppressor genes in A549, H1975 and SPC-A1 (Figure 6D, 6E and Supplementary Figure S3A). Our results determined that NCALD was the most upregulated in response to linc00673 downregulation and siRNA could affect its transcript expression level. H1703 cells transfected with pcDNA3.1-linc00673 exhibited a decrease in NCALD expression relative to controls (Figure 6G). Furthermore, western blot analysis determined that the protein expression levels of NCALD in NSCLC cells after transfection were consistent with the results of qRT-PCR (Figure 6F, 6H and Supplementary Figure S3B).

### Linc00673 silences NCALD transcription by binding with Histone demethylase lysine specific demethylase 1 (LSD1)

Previous studies indicate that lncRNAs silence key downstream mediators through RNA binding proteins, such as PRC2, STAU1, and LSD1 [26–28]. In order to further investigate whether lin00673 inhibits NCALD expression through a similar mechanism, we predicted that the probabilities interaction of linc00673 and RNA binding protein via RNA-Protein interaction prediction website (http://pridb.gdcb.iastate.edu/RPISeq/) [29]. As presented in Figure 6I and Supplementary Figure S3C, the probabilities interaction of linc00673 with LSD1, SUZ12 and EZH2 were 0.7, 0.85, and 0.6 using RF classifier, however, these were 0.83, 0.85, and 0.88 using SVM classifier, respectively. Importantly, qRT-PCR and western blot analyses show that LSD1 knockdown could induce expression levels of NCALD mRNA and protein (Figure 6J, 6K and Supplementary Figure S3D), while, downregulation of EZH2 or SUZ12 expression do not increase NCALD mRNA expression (Supplementary Figure S3E). Based on these observations, we propose that linc00673 may reduce NCALD transcription through binding LSD1 in the NCALD promoter region. To validate this assumption, we first performed RIP analysis to validate accuracy of the prediction. Results indicated that linc00673 could directly bind LSD1 in A549 and H1975 cells (Figure 6L). Moreover, result of RNA pulldown also indicated that linc00673 interacted with LSD1 (Figure 6M). We then used ChIP to confirm the relationship between LSD1 and NCALD gene and results determined that LSD1 could directly bind the promoter region of the NCALD gene, and knockdown of linc00673 reduced LSD1-mediated H3K4me2 demethylation (Figure 6N).

### Decreases in NCALD may be involved in the oncogenic activation of linc00673

**Figure 7:**
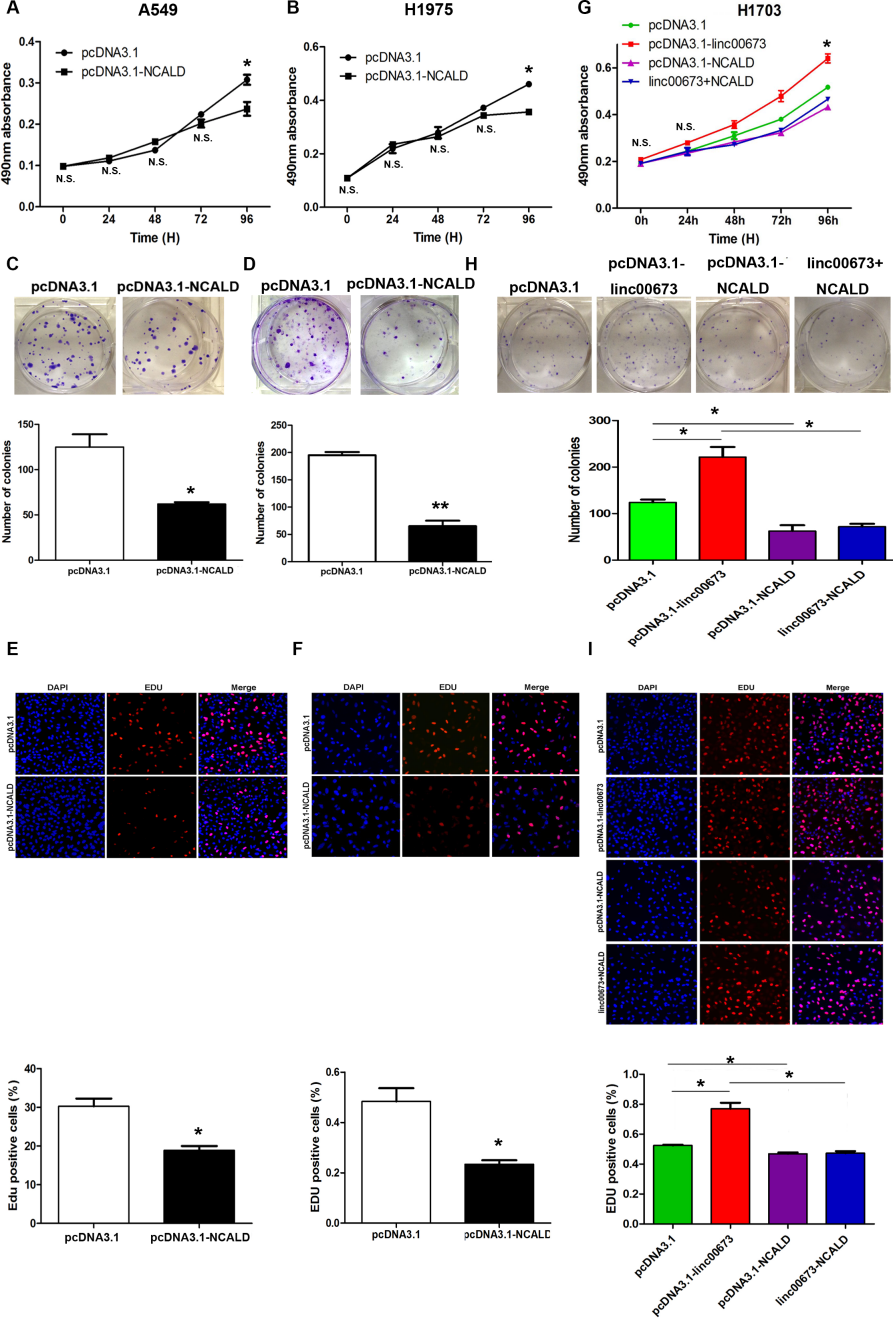
The effects of NCALD on NSCLC cell viability *in vitro* A549 and H1975 cells were transfected with pcDNA3.1 (control) and pcDNA3.1-linc00673. While H1703 cells were transfected with pcDNA3.1, pcDNA3.1-linc00673, pcDNA3.1-NCALD, linc00673 + NCALD. (**A**, **B** and **G**) MTT assays were performed to determine the cell viability of transfected NSCLC cells. (**C**, **D** and **H**) A colony-forming assay was conducted to determine the proliferation of transfected NSCLC cells. The colonies were counted and captured. (**E**, **F** and **I**) EDU (red)/DAPI (blue) immunostaining assay was used to confirme the results of MTT assay and colony-forming assay. The data represent the mean ± SD from three independent experiments. *P < 0.05, **P < 0.01.

To determine the role of NCALD in NSCLC cell proliferation, we carried out gain-of-function assays. After transfection with a pcDNA3.1-NCALD vector, NCALD expression levels were significantly upregulated in A549, H1975, SPC-A1 cells (Supplementary Figure S3F). Using MTT, EDU and colony formation assays, we found that overexpression of NCALD could inhibit the NSCLC cells ability to proliferate (Figure 7A–7F and Supplementary Figure S3H–S3J). Furthermore, we performed rescue experiments with H1703 cells to investigate whether NCALD was implicated in linc00673 induced cell proliferation. Results from western blot analysis revealed that NCALD expression levels were upregulated in pcDNA3.1-NCALD and linc00673+NCALD groups (Supplementary Figure S3G). Presented in Figure 7G, 7H and 7I, NCALD overexpression partially compromised the effects of linc00673 on NSCLC proliferation. We conclude that linc00673 promotes NSCLC cell proliferation through downregulation of NCALD.

### NCALD expression was downregulated in cancer tissues and low NCALD level is associated with poor prognosis in NSCLC patients

**Figure 8:**
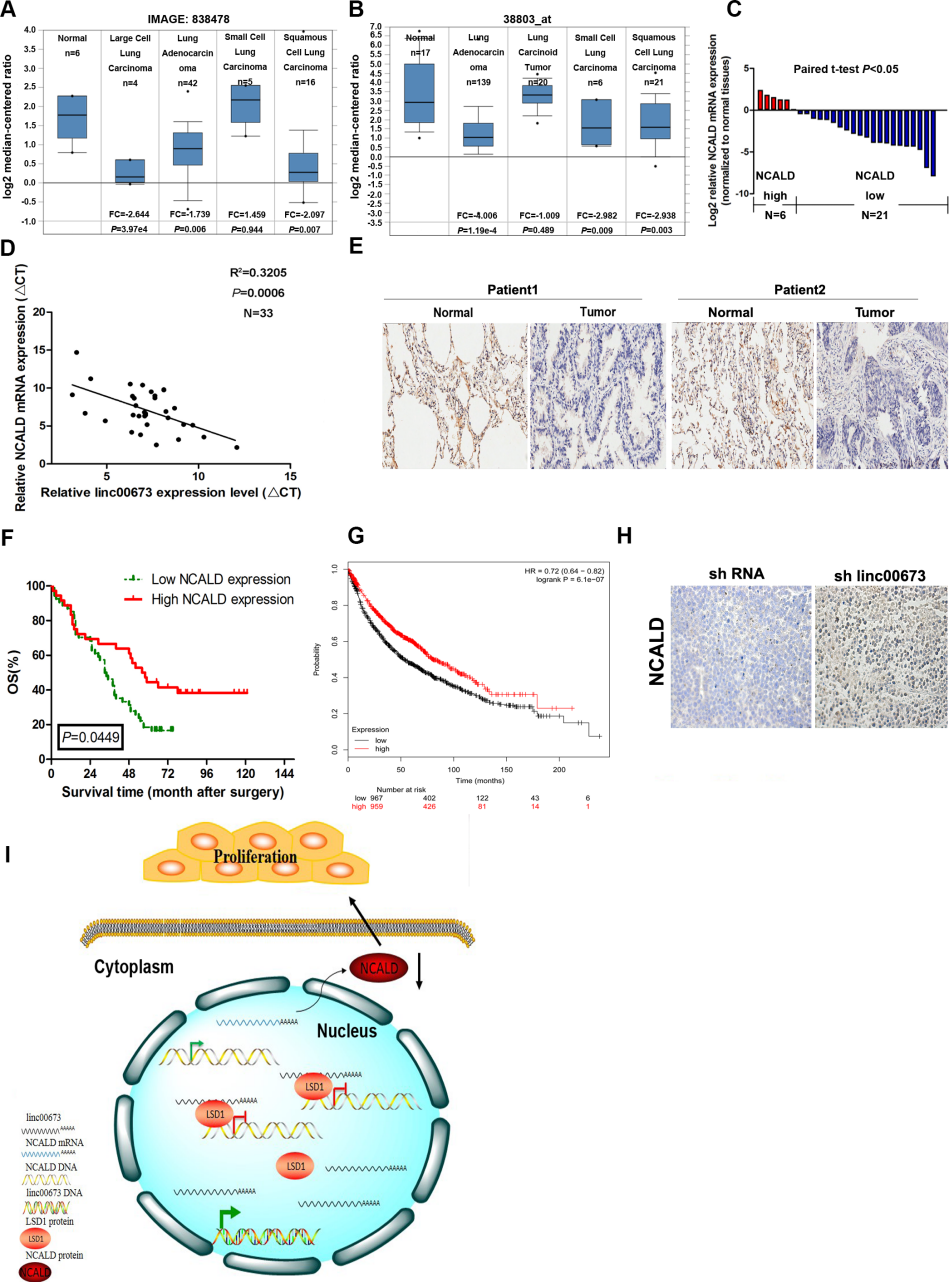
The NCALD expression level in NSCLC tissues and the clinical significance of NCALD (**A** and **B**) Selected datasets from the oncomine cancer microarray database were mined to determine the alterations of NCALD in mRNA expression levels. Representative datasets from Garber and Bhattacharjee lung cancer cohorts. (**C**) QRT-PCR analysis of NCALD mRNA expression level in NSCLC tissues. (**D**) Analysis of the relationship between linc00673 expression (ΔCt value) and NCALD mRNA level (ΔCt value) in 33 NSCLC tissues. (**E**) Immunohistochemical staining of normal and NSCLC tissues with anti-NCALD antibody. Representative two patient samples are shown. 90 patient samples are stained and analyzed. (**F**) Kaplan–Meier analysis of OS based on NCALD expression in all 90 patients. The patients were divided into a low NCALD expression group (*n* = 35) and a high NCALD expression group (*n* = 55) according to whether their samples showed up or downregulation of NCALD. (**G**) Kaplan–Meier survival plots demonstrating the good prognostic effect of NCALD downregulation correlated with a worse OS in lung cancer patients (*n* = 1926). (**H**) Immunohistochemical staining of tumor tissues formed from shRNA linc00673 or empty vector transfected A549 cells with anti-NCALD antibody. Representative images are shown. (**I**) Summary diagram describes that NCALD regulates NSCLC cell proliferation. *P < 0.05, **P < 0.01.

To establish the clinical significance of NCALD, we carried out an expression analysis of NCALD using two microarray data sets from Garber and Bhattacharjee lung cancer cohorts downloaded from Oncomine [30–31]. In both cohorts, the expressionof NCALD mRNA was significantly lower in NSCLC tissues compared to normal tissues (Figure 8A and 8B). Furthermore, we also examined the expression level of NCALD mRNA in paired clinical NSCLC tissues and adjacent normal tissues. Results indicated that NCALD mRNA was downregulated in NSCLC tissues, in agreement with the data from Oncomine (Figure 8C), and the NCALD mRNA expression level was inversely correlated with linc00673 in these samples (Pearson's correlation coefficient *r*^2^ = 0.3205, *P* = 0.0006, Figure 8D). We consistently found that NCALD protein abundance is decreased in NSCLC samples using tissue microarrays (TMAs) to analyze 90 primary operable NSCLC cases (Figure 8E). High NCALD protein expression was significantly associated with increased survival overall (OS; *P* = 0.0449, Figure 6F). Finally, the positive prognostic effect of NCLAD expression was also supported by Kaplan–Meier Plotter analysis (www.kmplot.com), which indicated that higher NCALD abundance correlated with a better OS, using microarray data from 1926 lung cancer patients [32] (Figure 8G). Moreover, the expression level of NCALD protein is upregulated in shRNA linc00673-transfected tumors (Figure 8H).

All together, these results validate that the expression levels of NCALD inversely correlated with linc00673 level in NSCLC tissues and low NCALD level was associated with NSCLC progression.

## DISCUSSION

Our knowledge about the gene-regulatory mechanisms that govern the progress of NSCLC is still considered fragmentary. Before the discovery of lncRNA, previous studies had shown that the mutations in certain protein-coding genes (such as TP53, EGFR, KRAS) are critical for the pathogenesis of NSCLC [5, 33]. However, recent searches for cancer drivers are focused on the important functions of lncRNA. In the present study, we revealed a novel transcript linc00673 through bioinformatics. Linc00673 is encoded at 17q25.1 and is approximately 2275 nt in length. Although previous studies have also identified linc00673 was upregulation in lung cancer tissues [34–35], very little is known about biological significance and the molecular mechanisms of linc00673 in NSCLC carcinogenesis. To this end, we used loss of function and gain of function assays to connect linc00673 to the promotion of cell proliferation. It has been reported that human NCALD gene was abundantly expressed in brain but its expression could also be detected in other tissues, such as natural killer cells, lymphoblasts, testis and ovary [20]. In our study, we found that NCALD was downregulated in NSCLC tissues and related with patients' prognosis. These findings suggest that linc00673 plays an oncogenic role in NSCLC progression by epigenetic regulation of NCALD expression. A growing body of evidence has documented the critical roles of lncRNAs and RNA-binding proteins (RBPs) in human cancers. For example, all three well characterized lncRNAs, HOTAIR, ANRIL and FAL1, can bind to PRC complexes resulting in gene transcription regulation. Tsai et al. have demonstrated that HOTAIR interacts with PRC2 and LSD1 to exert its functional role in epigenetically regulating gene expression and promoting human tumor metastasis [27, 36]. It has been documented that ANRIL recruits PRC1 [37] and PRC2 [38] to corresponding gene promoter regions leading to repression the transcription of the tumor suppressor gene. Hu et al. found that FAL1 associates with the BMI1, an epigenetic repressor, and reduced a number of gene transcrips including CDKN1A [39]. Here, we extend the previous observations of lncRNA interaction with RBPs and provided evidence that the functional interaction with chromosome modification complexes might be a predominant molecular mechanism by which lncRNA exerts its biological function.

Histone modifications, including methylation, are considered important factors in transcriptional activation and repression [40]. Perturbations in the balance of histone methylation and demethylation could impact gene expression and contribute to human cancer. LSD1, also known as KDM1A, is the histone demethylase that specifically demethylates mono- and dimethylated lysine 4 of histone H3 (H3K4) [41]. It is well documented that LSD1 is necessary for mammalian development and participates in many biological processes, such as cell differentiation and cell cycle progression [42–43]. In our previously studies, we indicated that LSD1 downregulation inhibits tumor cell proliferation [44]. However, the precise regulatory mechanism for LSD1 in NSCLC remains largely unknown. Interestingly, in this study, through RNA transcriptome sequencing and qRT-PCR assays, we found NCALD was remarkably upregulated after linc00673 knockdown. Additionally, our results showed that LSD1 silenced NCALD expression by epigenetic regulation. Based on the collective results presented above, we propose that linc00673 exerts its oncogenic function, at least in part, via binding LSD1 and inhibiting NCALD expression in NSCLC tumorigenesis (Figure 8I).

Since continuing advances in transcriptomics demonstrate that lncRNAs fulfill key functions in the regulation of gene expression, and lncRNAs are exceedingly deregulated in human cancer [6], it is believed that lncRNA might be an important supplement to proteins and other effectors in complex regulatory networks of human cancers. Our findings in this study provide a plausible molecular mechanism underlying the deregulation of cancer-associated lncRNA expression in NSCLC. Our study explores the functional role and mechanism of linc00673 in cell proliferation. In consideration of the relationship between linc00673 expression level and lymphatic metastasis, whether linc00673 playsa vital role in NSCLC cell metastasis and its relevant mechanisms remains to be clarified. Only with full elucidation of linc00673 functionality and molecular mechanisms relevant to NSCLC, can we open avenues for the use of lncRNAs in identification and treatment of novel diagnostic or predictive biomarkers and targets.

## MATERIALS AND METHODS

### Gene expression datasets

Lung cancer gene expression profile was obtained from the NCBIs Gene Expression Omnibus (GEO, http://www.ncbi.nlm.nih.gov/geo/). GSE18842 is microarray data that studied 91 samples, including 46 lung tumors samples (14 adenocarcinomas and 32 squamous-cell carcinomas) and 45 corresponding nontumor samples. The gene expression profiles of all lung tissue samples were analyzed by the Human Genome U133 Plus 2.0 chip from Affymetrix. We re-annotated the microarray using blast +2.2.30 after downloading probe sequences from GEO or microarray manufacturers. For multiple probes corresponding to one lncRNA, maximum normalized signal was selected.

lncRNA expression profiles of lung tumors were generated using significance analysis of microarrays (SAM) in MeV software (http://www.tm4.org/mev.html). The threshold set for up- or down-regulated genes was a fold change ≥ 2 or ≤ 0.5. In addition, we controlled the delta value so that median number of false significant genes was set at zero. Two-class paired or two-class unpaired analyses were used according to experimental design.

### Patients and tissue samples

We obtained paired NSCLC and adjacent normal lung tissues from 80 patients who underwent primary surgical resection in the Department of Thoracic Surgery, Jinling Hospital, Nanjing University School of Medicine, China, between February 2013 and July 2015. No patient had received local or systemic treatment before any operation. All collected tissue samples were immersed in RNA Later stabilization solution (Qiagen, Hilden, Germany) and were immediately frozen in liquid nitrogen and stored at −80°C until RNA isolation. The clinicopathologic characteristics of the patients with NSCLC, including tumor size, lymph node metastasis and TNM, were recorded and summarized in Table 1. Our study protocol was approved by the Institutional Review Board of Nanjing University, and all of the participants signed an informed consent form.

### Cell culture

Five NSCLC adenocarcinoma cell lines (A549, SPC-A1, PC9, H1299 and H1975) and three NSCLC squamous carcinomas cell lines (H1703, H520 and H226) were purchased from the Institute of Biochemistry and Cell Biology of the Chinese Academy of Sciences (Shanghai, China) in May, 2014. The cells were obtained within 6 months directly from The Chinese Academy of Sciences cell bank. All cell lines have a short tandem repeat profiling characterization in The Chinese Academy of Sciences. A549, H1299, H1703, H520, H1975, H226 cells were maintained in RPMI Medium 1640 basic media (GIBCO-BRL, Invitrogen, Carlsbad, CA), and SPCA1, PC9, HBE cells were grown in Dulbecco's modified Eagle's media (DMEM; GIBCO-BRL, Invitrogen) in a humidified incubator at 37°C with 5% CO_2_. All of the media used was supplemented with heat-inactivated 10% fetal bovine serum (FBS) and antibiotics (100 U/ml penicillin and 100 mg/ml streptomycin) (Invitrogen, Carlsbad).

### RNA isolation and quantitative reverse transcriptase polymerase chain reaction (qRT-PCR) analyses

Total RNA extraction and qRT-PCR analyses were performed as previously described [17]. The specific primer sequences used are shown in Supplementary Table S1 Sheet 1.

### 3′-Race and 5′-Race

5′ and 3′ RACE were performed using the First Choice-RLM RACE kit according to manufacturers' instructions (Life Technologies).

### RNA interference by siRNA

Twenty-four hours after cells were plated in six-well plates, the small interfering RNA (siRNA) and nonspecific control siRNA was (Carlsbad, California, USA) transfected into cells using Lipofectamine 2000 (Invitrogen, Shanghai, China), according to the manufacturers' instructions. Sequences of siRNAs are described in Supplementary Table S1 Sheet2. Cells were harvested 48 h after transfection for qRT-PCR and Western blot analysis.

### Plasmid DNA transfection

The linc00673 sequence was synthesized according to the full-length linc00673 sequence lacking a poly A tail (based on the linc00673 sequence, NR_036488.1, in NCBI). The NCALD sequence was synthesized according to the CDS sequence of NCALD. Both were subcloned into a pcDNA3.1 vector (Invitrogen, Shanghai, China). Plasmid vectors (pcDNA3.1-linc00673, pcDNA3.1-NCALD and empty vector) were transfected into NSCLC cells cultured in six-well plates using X-treme GENE HP DNA transfection reagent (Roche, Basel, Switzerland), according to the manufacturers' instructions. The empty vector was used as the control. Cells were harvested 48 h after transfection for qRT-PCR and Western blot analysis.

### MTT assay and colony formation assay

The scramble, or si-linc00673 transfected A549, SPC-A1 or H1975 cells (3,000/well) and empty vector- or pcDNA3.1-linc00673-transfected H1703 cells (3,000/well), were allowed to grow in 96-well plates. Cell viability was documented every 24 h following the manufacturers' protocol. All experiments were performed in quadruplicate. For the cells to form colonies, a total of 700 transfected cells and control cells were placed onto a fresh six-well plate and maintained in media containing 10% FBS, media was replaced every 4 d. After 2 wk, the colonies were fixed with methanol and stained with 0.1% crystal violet (Sigma, St. Louis, MO). Visible colonies were manually counted. Triplicate wells were assessed for each treatment group.

### Flow-cytometric analysis of cell cycle

For the flow-cytometric analysis of the cell cycle, the transfected cells were stained with Propidium iodide (PI) using the CycleTEST TM PLUS DNA reagent kit (BD Biosciences) following the protocol and analyzed by FACScan. The percentage of cells in the S, G0/G1, and G2/M phases were counted and compared.

### Ethynyldeoxyuridine (EDU) analysis

5-ethynyl-2-deoxyuridine (EDU) labeling/detection kit (Ribobio, Guangzhou, China) was used to assess the cell proliferation. Cells were grown in 96-well plates at 5 × 103 cells/well. Forty-eight hours after transfection, 50 μM EdU labeling media was added to the 96-well plates and they were incubated for 2 h at 37°C under 5% CO2. After treatment with 4% paraformaldehyde and 0.5% Triton X-100, cells were stained with anti-EdU working solution. DAPI was used to label cell nuclei. The percentage of EdU-positive cells was calculated after analyses of fluorescent microscopy. Five fields of view were randomly assessed for each treatment group.

### Isolation of cytoplasmic and nuclear RNA

Cytoplasmic and nuclear RNA were separated and purified using the PARIS Kit (Life Technologies, Carlsbad, CA, USA) according to the manufacturers' instructions.

### Tumor formation assay in a nude mouse model

Animal experiments were performed as previously described (17). Male and 4 wkathymic BALB/c nude mice were used for the tumor formation assay. A549 cells transfected with shRNA linc00673 or an empty vector were cultured in six well plates for 48 h. Then, the cells were washed with PBS and resuspended at a concentration of 2 × 107/ml. Each mouse was injected on one side of the posterior flank with 100 ul of cell suspension. Tumor growth was measured using calipers every 3 d. The tumors were removed from all animals after 14 d, and the subcutaneous growth of each tumor was examined. Tumor volumes were calculated using the equation V = 0.5 × D × d2 (V, volume, D: longitudinal diameter, d: latitudinal diameter). Animal care and experimental procedures were approved by the Model Animal Research Center of Jingling Hospital and conducted according to Institutional Animal Care and User guidelines. All of the surgeries were performed under sodium pentobarbital anesthesia, and all efforts were made to minimize suffering.

### RNA-seqbioinformatic analysis

The mRNA-Seq experiments were performed by Novogene (Beijing, China). mRNA-seq library was prepared for sequencing using standard Illumina protocols. Briefly, total RNAs from scrambled, or si linc00673 transfected A549 cells, were isolated using TRIzol reagent (Invitrogen). To remove any contaminating genomic DNA, the total RNA was treated with RNase-free DNase I (New England Biolabs, MA, USA). mRNA extraction was performed using Dynabeadsoligo(dT) (Invitrogen Dynal). Superscript II reverse transcriptase (Invitrogen) and random hexamer primers were used to synthesize double-stranded complementary DNAs. To create the mRNA-seq library, the cDNAs were then fragmented by nebulization and the standard Illumina protocol followed. For the data analysis, basecalls were performed using CASAVA. Reads were aligned to the genome using the split read aligner TopHat (v2.0.7) and Bowtie2, using default parameters. HTSeq was used for estimating abundances.

### Protein analysis

Forty-eight hours post-transfection, cells were lysed using a lysis buffer containing the mammalian protein extraction reagent RIPA (Beyotime China), a protease inhibitor cocktail (Roche, Basel, Switzerland) and PMSF (Roche). Cells' protein lysates that contained 50 μgprotein were electrophoresed on 10% SDS-PAGE and transferred onto 0.22 mm NC membranes (Sigma–Aldrich), and incubated with specific antibodies. Specific bands were detected by ECL chromogenic substrate and quantified by densitometry (Quantity One software, BioRad). GAPDH antibody was used as control. Anti-CDK2, CDK4, CDK6 (1:1,000) were purchased from Cell Signaling Technology, Inc., anti-NCALD was purchased from Proteintech.

### Immunohistochemistry study

All NSCLC tissues and associated normal lung tissues were fixed in 10% formalin and embedded in paraffin. Tissues were then stained with immunohistochemistry using the following antibody: NCALD (1:50, Proteintech) following cut at a thickness of 4 μm. Immunoreaction was performed using the labeled streptavidin-biotin method with overnight incubation. Diaminobenzidine was used for visualization. To quantify NCALD protein expression, both the intensity and extent of immunoreactivity were evaluated and scored in randomly selected five representative fields of vision at medium magnification. The resulting score was calculated by adding up the staining intensity (0 = no staining, 1 = mild staining, 2 = moderate staining and 3 = strong staining) and the percentage of positive cells (0 was ≤ 5% positive, 1 was 6%–25% positive, 2 was 26%–50% positive, 3 was 51%–75% positive and 4 was > 75% positive). Immunostaining was considered negative (or −) when the score was 0–1, weak positive (or 1+) when the score was 2–3, medium positive (or 2+) when the score was 4–5 and strong positive (or 3+) when the score was 6–7.

### RNA binding protein immunoprecipitation (RIP) assay

RNA immunoprecipitation (RIP) experiments were performed by using a Magna RIP RNA-Binding Protein Immunoprecipitation Kit (Millipore, USA) according to the manufacturer's instructions. Antibody for RIP assays of LSD1 were purchased from Millipore. In brief, cells were lysed and incubated with protein A Sepharose beads which were conjugated with antibodies at 4°C. After 3–6 hours, the beads were washed with wash buffer and then the complexes digest the proteins using a heated water bath with 0.1% SDS and 0.5 mg/mL Proteinase K (30 minutes at 55°C). Finally, immunoprecipitated RNA was purified and analyzed via qRT-PCR.

### RNA pulldown assay

Linc00673 was transcribed from vector pcDNA3.1-linc00673 *in vitro*. The Biotin RNA Labeling Mix (Roche Diagnostics, Indianapolis, IN) and T7/SP6 RNA polymerase (Roche Diagnostics, Indianapolis, IN) were used for RNA biotin-label, and RNeasy Mini Kit (Qiagen, Valencia, CA) was used for purification. Next, 1 milligram whole-cell lysates from A549 cells was mixed with 3 μg of purified biotinylated transcripts and incubated with streptavidin agarose beads (Invitrogen) for 1 h at 25°C. The beads were washed briefly and boiled in sodium dodecyl sulfate (SDS) buffer, and the retrieved protein was analyzed by the standard western blot technique.

### Chromatin immunoprecipitation assays

EZ-CHIP KIT (Millipore, USA) was used to perform the chromatin immunoprecipitation assays. First, in order to generate DNA–protein cross-links, A549 and H1975 cells were incubated with formaldehyde. Cells were then lysed, sonicated and immunoprecipitated with LSD1 and H3K4me2-specific antibody (Millipore, USA) or IgG, as control. Precipitated chromatin DNA was recovered and analyzed by qPCR. The sequences of ChIP primers for NCALD promoter region amplification are shown in Supplementary Table S1 Sheet1.

### Statistical analysis

All statistical analyses were performed using SPSS 17.0 software (IBM, Chicago, IL, USA) and differences were considered to be statistically significant at *P* < 0.05. Student's *t*-test (two-tailed), chi-square test analysis and Mann–Whitney tests were performed to analyze differences between groups. Pearson correlation analyses were used to investigate the correlation between linc00673 and NCALD mRNA expression. Kaplan–Meier method with the log-rank test wasapplied to calculate overall survival (OS) rates.

## SUPPLEMENTARY MATERIALS TABLE AND FIGURES




