# Supplementary material for: Upregulation of long intergenic noncoding RNA 00673 promotes tumor proliferation via LSD1 interaction and repression of NCALD in non-small-cell lung cancer
Source: Oncotarget. 2016 Mar 24;7(18):25558–75. doi: 10.18632/oncotarget.8338 (PMC5041926; doi:10.18632/oncotarget.8338)
Supplement: Supplementary file 1 [file oncotarget-07-25558-s001.pdf]

Upregulation of long intergenic noncoding RNA 00673 promotes tumor proliferation via LSD1 interaction and repression of NCALD in non-small-cell lung cancer

Supplementary Materials

Supplementary Table S1: (Sheet1) The specific primer sequences used were listed. (Sheet2) The sequences of siRNAs used were listed. (Sheet3) The list of 27 upregulation lncRNAs and 72 downregulation lncRNAs analysed by microarray dataset GSE18842. (Sheet4) The list of 499 downregulation transcripts and 489 upregulation transcripts through RNA transcriptome sequencing analysis in A549 cells following linc00673 knockdown.

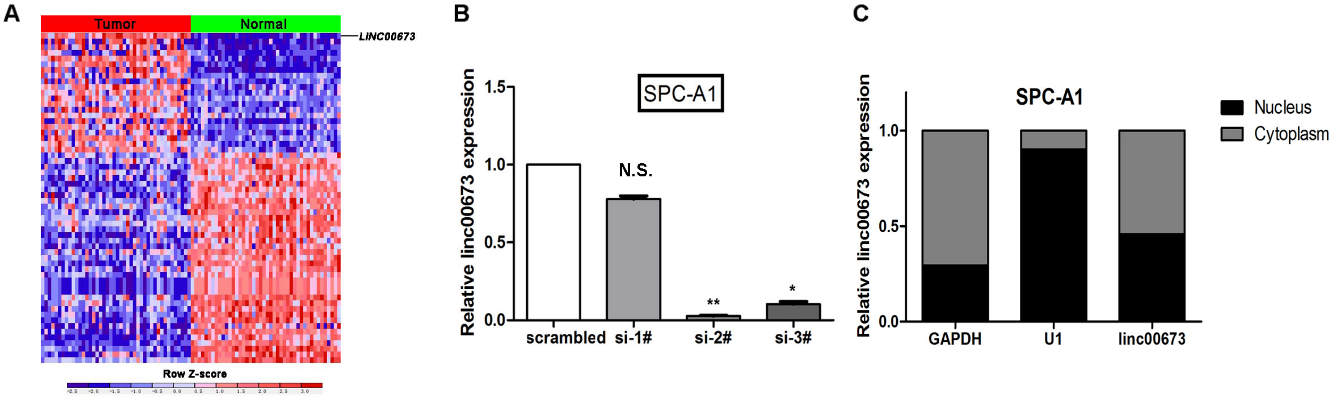

Supplementary Figure S1: (**A**) A heatmap representation of the lncRNAs with the upregulation and downregulation across NSCLC and normal lung tissues in GSE18842. (**B**) qRT-PCR analysis of linc00673 expression level in SPC-A1 transfected with three discrete chemically synthesized siRNAs. (**C**) Linc00673 expression levels in different subcellular fractions inSPC-A1 cells were detected by qRT-PCR. Black range indicates nuclear fraction; Gray indicates cytoplasmic fraction. The data represent the mean ± SD from three independent experiments. *$P < 0.05$, **$P < 0.01$.

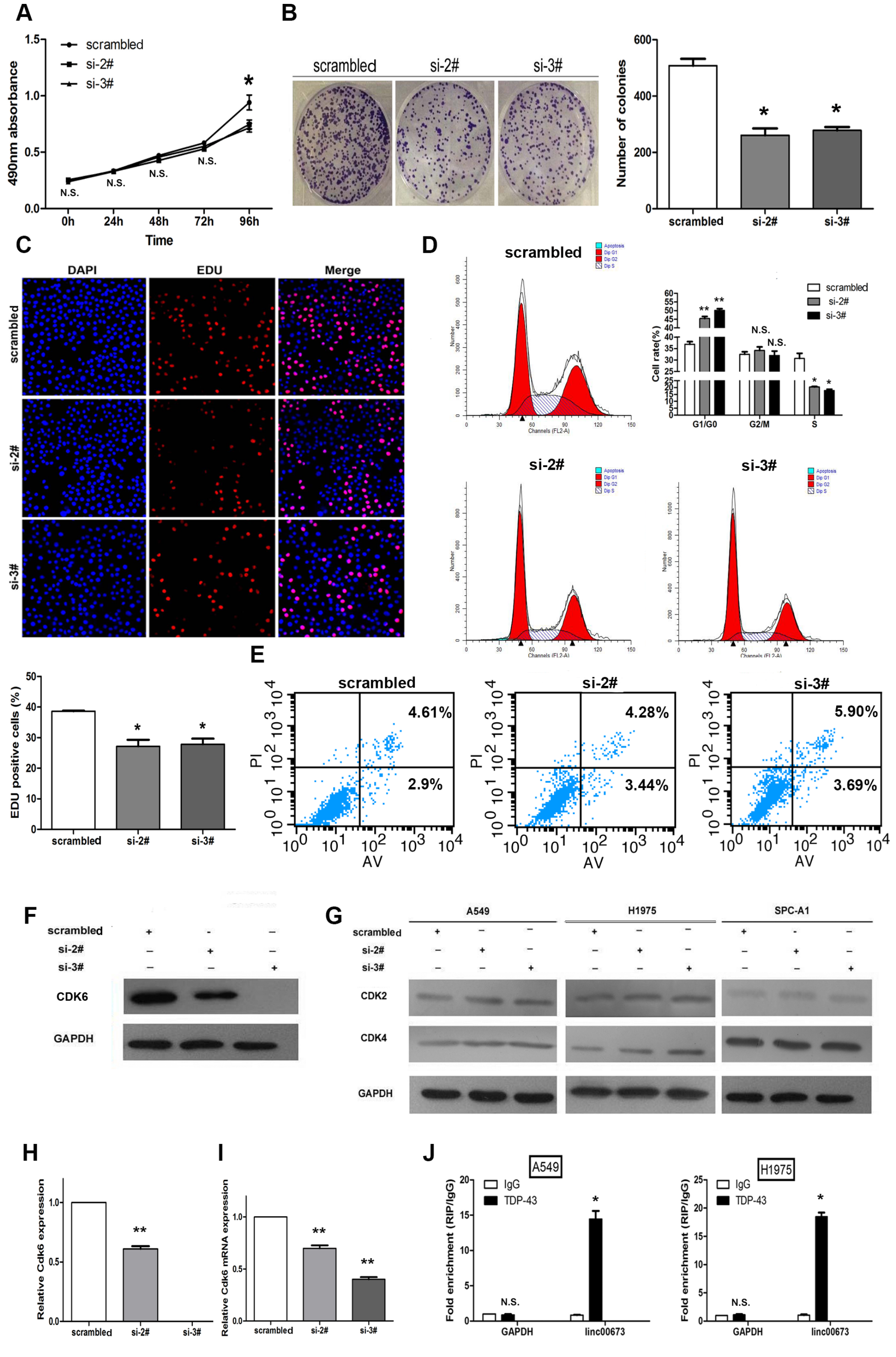

Supplementary Figure S2: (**A**) MTT assays were performed to determine the cell viability of siRNA linc00673 (si-2# or si-3#) transfected SPC-A1 cells. (**B**) A colony-forming assay was conducted to determine the proliferation of siRNA linc00673 (si-2# or si-3#) transfected SPC-A1 cells. The colonies were counted and captured. (**C**) EDU (red)/DAPI (blue) immunostaining assay was used to confirme the results of MTT assay and colony-forming assay. (**D**) Cell cycle analyses in the siRNA linc00673 (si-2# or si-3#) transfected SPC-A1 cell lines. Representative fluorescence activated cell sorting images and statistics based were presented. (**E**) FACS technology was used to determine the effect of linc00673 on apoptosis. (**F** and **H**) Analysis of CDK6 protein level in SPC-A1 cells by Western blot. GAPDH protein expression was used as an internal. (**G**) qRT-PCR analysis of CDK2 and CDK4 mRNA expression level in A549, H1975, and SPC-A1 cells. (**I**) qRT-PCR analysis of CDK6 mRNA expression level in SPC-A1 cells. (**J**) RIP with rabbit monoclonal anti-TDP-43 and preimmune IgG from A549 (left) and H1975 (right) cell extracts. RNA levels in immunoprecipitates were determined by qPCR. Expression levels of linc00673 RNA were presented as fold enrichment in TDP-43 relative to IgG immunoprecipitates. The data represent the mean ± SD from three independent experiments. $*P < 0.05$, $**P < 0.01$.

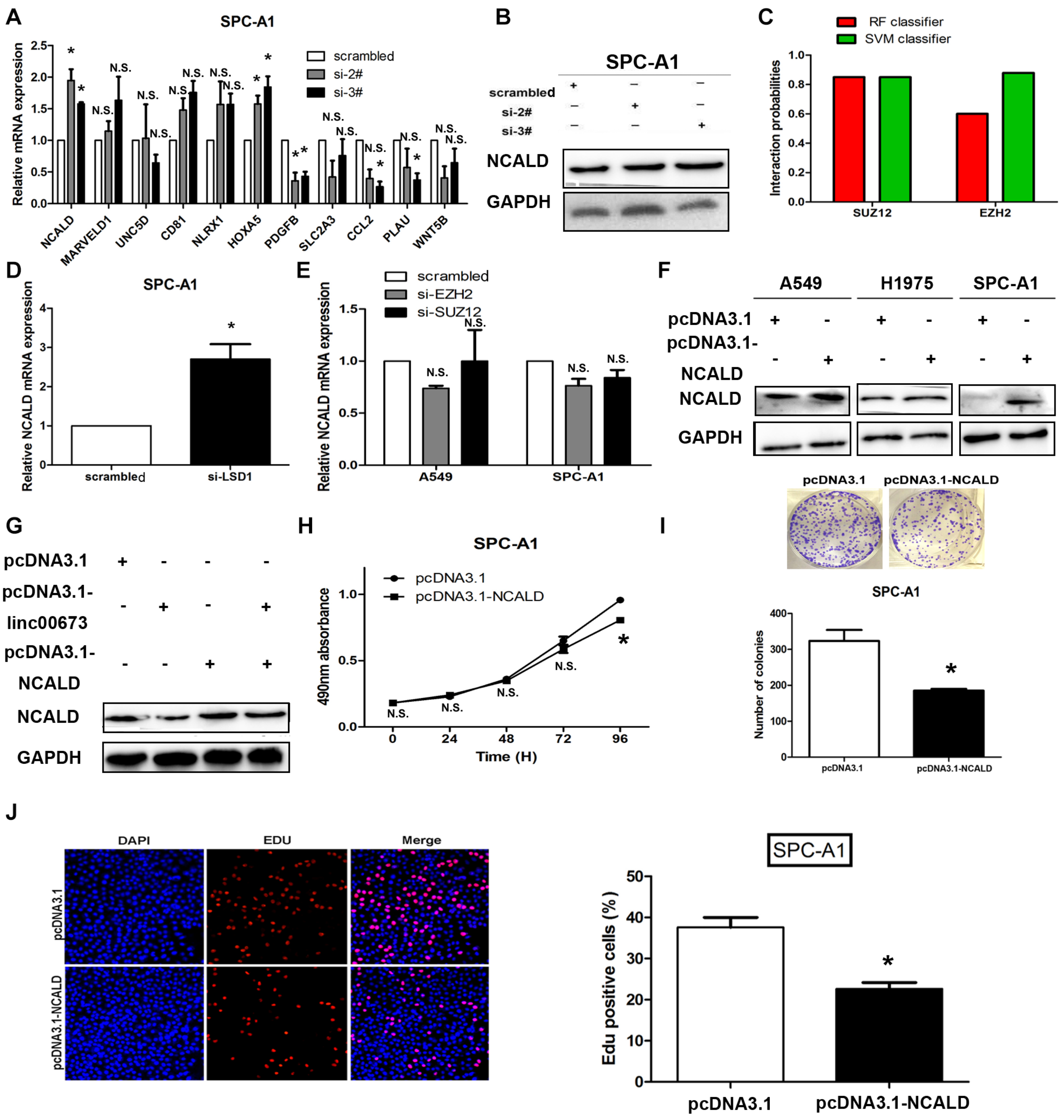

Supplementary Figure S3: (**A**) QRT-PCR analysis of the mRNA expression levels of tumor suppressor genes and oncogenes in control (scrambled) vs siRNA linc00673 (si-2# and si-3#) -treated SPC-A1 cells. (**B**) Western blot analysis of NCALD protein level in SPC-A1 cells. GAPDH protein expression was used as an internal control. (**C**) The prediction of the interaction probabilities of linc00673 with RNA binding protein (EZH2 and SUZ12) via RNA-Protein interaction prediction (http://pridb.gdcb.iastate.edu/RPISeq/). (**D**) qRT-PCR analysis of NCALD mRNA expression level in si-LSD1 transfected SPC-A1 cells. (**E**) qRT-PCR analysis of NCALD mRNA expression level in si-EZH2 or si-SUZ12 transfected A549 and SPC-A1 cells. (**F**) Western blot analysis of NCALD protein level in pcDNA3.1-NCALD or pcDNA3.1 transfected A549, H1975 and SPC-A1 cells. GAPDH protein expression was used as an internal. (**G**) Western blot analysis of NCALD protein level in pcDNA3.1, pcDNA3.1-NCALD or pcDNA3.1-linc00673 transfected H1703 cells. (**H**) MTT assays were performed to determine the cell viability of pcDNA3.1 or pcDNA3.1-NCALD transfected SPC-A1 cells. (**I**) A colony-forming assay was conducted to determine the proliferation of pcDNA3.1 or pcDNA3.1-NCALD transfected SPC-A1 cells. The colonies were counted and captured. (**J**) EDU (red)/DAPI (blue) immunostaining assay was used to confirme the results of MTT assay and colony-forming assay. The data represent the mean ± SD from three independent experiments. $^*P < 0.05$, $^{**}P < 0.01$.
